# Genomic analysis for managing small and endangered populations: a case study in Tyrol Grey cattle

**DOI:** 10.3389/fgene.2015.00173

**Published:** 2015-05-13

**Authors:** Gábor Mészáros, Solomon A. Boison, Ana M. Pérez O'Brien, Maja Ferenčaković, Ino Curik, Marcos V. Barbosa Da Silva, Yuri T. Utsunomiya, Jose F. Garcia, Johann Sölkner

**Affiliations:** ^1^Division of Livestock Sciences, University of Natural Resources and Life SciencesVienna, Austria; ^2^Department of Animal Science, University of ZagrebZagreb, Croatia; ^3^Empresa Brasileira de Pesquisa AgropecuáriaJuiz de Fora, Brazil; ^4^UNESP-Universidade Estadual PaulistaJaboticabal, Brazil

**Keywords:** breed management, endangered breeds, SNP chip, linkage disequilibrium, runs of homozygosity, genomic selection

## Abstract

Analysis of genomic data is increasingly becoming part of the livestock industry. Therefore, the routine collection of genomic information would be an invaluable resource for effective management of breeding programs in small, endangered populations. The objective of the paper was to demonstrate how genomic data could be used to analyse (1) linkage disequlibrium (LD), LD decay and the effective population size (Ne_LD_); (2) Inbreeding level and effective population size (Ne_ROH_) based on runs of homozygosity (ROH); (3) Prediction of genomic breeding values (GEBV) using small within-breed and genomic information from other breeds. The Tyrol Grey population was used as an example, with the goal to highlight the potential of genomic analyses for small breeds. In addition to our own results we discuss additional use of genomics to assess relatedness, admixture proportions, and inheritance of harmful variants. The example data set consisted of 218 Tyrol Grey bull genotypes, which were all available AI bulls in the population. After standard quality control restrictions 34,581 SNPs remained for the analysis. A separate quality control was applied to determine ROH levels based on Illumina GenCall and Illumina GenTrain scores, resulting into 211 bulls and 33,604 SNPs. LD was computed as the squared correlation coefficient between SNPs within a 10 mega base pair (Mb) region. ROHs were derived based on regions covering at least 4, 8, and 16 Mb, suggesting that animals had common ancestors approximately 12, 6, and 3 generations ago, respectively. The corresponding mean inbreeding coefficients (*F*_ROH_) were 4.0% for 4 Mb, 2.9% for 8 Mb and 1.6% for 16 Mb runs. With an average generation interval of 5.66 years, estimated Ne_ROH_ was 125 (Ne_ROH>16 Mb_), 186 (Ne_ROH>8 Mb_) and 370 (Ne_ROH>4 Mb_) indicating strict avoidance of close inbreeding in the population. The LD was used as an alternative method to infer the population history and the Ne. The results show a continuous decrease in Ne_LD_, to 780, 120, and 80 for 100, 10, and 5 generations ago, respectively. Genomic selection was developed for and is working well in large breeds. The same methodology was applied in Tyrol Grey cattle, using different reference populations. Contrary to the expectations, the accuracy of GEBVs with very small within breed reference populations were very high, between 0.13–0.91 and 0.12–0.63, when estimated breeding values and deregressed breeding values were used as pseudo-phenotypes, respectively. Subsequent analyses confirmed the high accuracies being a consequence of low reliabilities of pseudo-phenotypes in the validation set, thus being heavily influenced by parent averages. Multi-breed and across breed reference sets gave inconsistent and lower accuracies. Genomic information may have a crucial role in management of small breeds, even if its primary usage differs from that of large breeds. It allows to assess relatedness between individuals, trends in inbreeding and to take decisions accordingly. These decisions would be based on the real genome architecture, rather than conventional pedigree information, which can be missing or incomplete. We strongly suggest the routine genotyping of all individuals that belong to a small breed in order to facilitate the effective management of endangered livestock populations.

## Introduction

In the last decade, technological advancement has allowed for the genotyping of large numbers of single nucleotide polymorphisms (SNP) in the genome. The increase in SNP density was accompanied with decrease in price for the commercial SNP-chips, standard sets of SNPs selected, and sold by genotyping companies in large numbers, dominating animal, and plant breeding research in many countries.

Traditionally microsatellite markers were used for genotyping animals in population genetics studies. A popular set of microsatellites endorsed by the Food and Agriculture Organization (FAO) is widely used to evaluate genetic diversity in farm animals, especially endangered breeds (Baumung et al., 2004; Groeneveld et al., 2010). As a technological follow up, a set of SNPs could be used for a similar purpose. An advantage of the SNP markers is their occurrence on standard genotyping panels. Pooling of genotypes and comparison of different populations is feasible, contrary to the microsatellites, where (partially) different panels could be genotyped each time. The disadvantage of the current SNP panels from breed diversity perspective is their development in direction of commercial application in the most common species and breeds, with little research undertaken to prepare assays to replace the ISAG-FAO microsatellite panels.

The application of the SNP markers in animal breeding however, goes beyond population genetics. The early adopters were the large breeding organizations managing breeds with many animals and large financial capital. After the general success of the genomic selection approach (Meuwissen et al., 2001) the utilization of the genomic information has increased considerably. Today genomic breeding values (GEBV) are routinely used for making selection decisions, which has resulted in reducing the generation interval and increasing genetic gain compared to classical progeny testing systems in dairy cattle populations (Hutchison et al., 2014).

Genomic selection was an incentive to genotype nearly every young bull in many large cattle populations. This incentive is missing in smaller breeds because a large population size is generally perceived as a requirement to estimate reliable GEBV. Although there were numerous studies using SNP data in many small breeds, these are rather isolated efforts to demonstrate an interesting phenomenon or describe other interesting general aspect of a particular breed. Even though there are a relatively low number of animals to be genotyped in small populations, there is a general lack of routine genotyping in small breeds.

The objective of the paper is to demonstrate how genomic data could be used to ascertain population structure in small and endangered breeds, evaluate GEBV, and assess the range of potential applications from the breed management perspective. To tackle this goal, we have used the Tyrol Grey breed as an example to demonstrate some of the potential uses of genomic data in small and endangered populations. Some of the potential uses of genomic data are not applicable to the Tyrol Grey breed, but they are still extremely useful in the breed management context. We discuss such uses in the last part of the paper in order to give a comprehensive overview about the potential of genomics in small and endangered breeds.

## Material and methods

### Data and quality control

The Tyrol Grey cattle is an endangered cattle breed with population size of consisting of 3785 breeding animals as of 2013 (ÖNGENE, www.oengene.at, 2014). We were able to genotype all available sires due to its small population size. From the available 218 Tyrol Grey AI bulls, we have genotyped 99 animals with the Illumina® BovineSNP50 BeadChip (50 K) and 119 animals with the Illumina® BovineHD BeadChip (HD) with about 770 K SNPs.

Only the 49,394 SNPs appearing on both 50 K and HD chips were retained, as the 50 K chip is a standard genotyping platform used for routine genotyping in taurine cattle. SNP markers with unknown positions and those on sex chromosome were excluded.

Two separate quality checks of the data were undertaken. The first quality check was done and the data used for estimating linkage disequilibrium (LD), effective population size (Ne) and also in genomic prediction approaches. The second quality check followed the approach of Ferenčaković et al. (2013). This was a more stringent quality check to help reduce error that might occur when estimating genomic inbreeding from runs of homozygosity (ROH).

The first quality check of the available SNP data was undertaken with the following criteria. SNPs with call rate less than 90% and Hardy-Weinberg equilibrium Fishers's exact *P*-value below 10^−6^ were removed using PLINK v1.07 (Purcell et al., 2007). SNP markers with minor allele frequency (MAF) < 0.05 and those mapped to the same physical positions were also deleted. Samples with call rate lower than 90% were also discarded. After quality control, 34,581 SNPs remained. The second quality check has been applied in the calculation of ROH as we did not exclude SNPs with low MAF, with high LD or those deviating from HWE. Genotyping errors were reduced by discarding SNPs with Illumina GenCall score ≤ 0.7, SNPs with Illumina GenTrain score ≤ 0.4 and animals with more than 5% missing genotypes. The same quality control settings has been used in Ferenčaković et al. (2013). The analyses were based on 211 bulls each genotyped for the same 33,604 SNPs with average distance of 73.655 kb between adjacent SNPs (from 23 to 1,955,291 bp), all placed on 29 autosomes. ROH segments were identified as a part of the genome in which 15 or more consecutive homozygous SNPs at a density of one SNP on every 100 kb are not more than 1 Mb apart. ROH calculations were done using the algorithm implemented in SNP and Variation Suite (v7.6.8 Win 64; Golden Helix, Bozeman, MT, USA www.goldenhelix.com).

### Linkage disequilibrium

The squared correlation (*r^2^*) was used to measure the LD. The *r^2^* values were calculated using PLINK as pairwise comparisons of markers on the same chromosome, separated by less than 10 Mb. The decay of LD was analyzed using bins of 100 kb for the maximum distance between SNP pairs. Marker bins below 100 kb for a 50 K SNP panel generally generate very small numbers of pairwise LD values. PLINK calculates as *r^2^* between two SNPs:

$$r_{LD}^{2}={\left(\frac{\sum_{i\;=\;1}^{n}\;(g_{ij}-{\bar{g}}_{j})(g_{im}-{\bar{g}}_{m})}{\sqrt{\sum_{i\;=\;1}^{n}\;{\left(g_{ij}-{\bar{g}}_{j}\right)}^{2}}\;*\;\sqrt{\sum_{i\;=\;1}^{n}\;{\left(g_{im}-{\bar{g}}_{m}\right)}^{2}}}\right)}^{2}$$

Where *n* = number of individuals with non-missing genotype; *g* is the genotype allele count of 0, 1, and 2 for AA, AB, and BB, respectively for individual *i* of SNP *j* and SNP *m*.

The calculations of effective population size (Ne_LD_) were based on McEvoy et al. (2011). The Ne was based on the LD values:

$$E\left(r_{LD}^{2}\right)\approx \frac{1}{\alpha +4Ne_{LD}c}$$

Where *E*(*r*_LD_^2^) is the expected squared correlation of allele frequencies at a pair of loci, α is 2 when the impact of mutation is considered and 1 otherwise. Variable *c* is the genetic distance between loci in Morgans. The Ne was calculated as:

$$Ne_{LD}\approx \frac{1}{4c}*\left(\frac{1}{r_{LD}^{2}}-\alpha \right)$$

Assuming that the population has been constant in size, the approximation of Ne_LD_ is true for *t* generations ago, where *t* = 1.2*c* (Hayes et al., 2003). It has been noted that LD patterns from shorter inter marker distances were informative about the Ne in the more distant past, while markers separated by longer distances are informative about the recent Ne. The relationships describing the historical development of Ne_LD_ should be considered only as an approximation, as LD patterns might be affected by variety of factors (de Roos et al., 2008).

### Runs of homozygosity

The inbreeding coefficients (*F*_ROH_) were calculated from the formula proposed by McQuillan et al. (2008); *F*_ROH_ = *L*_ROH_/*L*_AUTOSOME_, where *L*_ROH_ is the total length of all ROH in the genome of an individual while *L*_AUTOSOME_ refers to the specified length of the autosomal genome covered by SNPs on the chip (here 2,499,624,571 bp). We calculated three coefficients; *F*_ROH>4 Mb_, *F*_ROH>8 Mb_, and *F*_ROH>16 Mb_ defined by the minimum ROH lengths being higher than 4, 8, or 16 Mb, respectively. Under the assumption that 1 cM = 1 Mb, calculated inbreeding coefficients are expected to correspond to the reference ancestral population being remote approximately 12 (*F*_ROH>4 Mb_), 6 (*F*_ROH>8 Mb_), and 3 (*F*_ROH>16 Mb_) generations. For more detailed explanation see Howrigan et al. (2011) and Curik et al. (2014).

The calculation of the effective population size (Ne_ROH_) was based on the equation Ne_ROH_ = 1/2ΔF, where ΔF was calculated as regression coefficient b, representing change of *F*_ROH>4 Mb_ per 1 year (regression of *F*_ROH>4 Mb_ on the birth year), multiplied by the generation interval 5.66, previously calculated from the Tyrol Grey pedigree by using the software ENDOG, version 4.6 (Gutiérrez and Goyache, 2005), together with pedigree inbreeding coefficient (*F*_PED_).

Autozygosity islands were defined as regions where SNPs had extreme ROH frequency (outliers according to boxplot distribution, see **Figure 6**).

Computation of descriptive statistics (PROC Means), bootstrap confidence intervals (SAS Macro), regression analysis (PROC REG), and graphical illustrations (PROC Boxplot, SAS Macro) were performed by the SAS software v 9.3.

### Genomic selection in small breeds

Single and multi-breed scenarios were considered to derive within and across breed GEBV. For the multi-breed scenarios the German-Austrian genotype pool of 6730 Fleckvieh and 1415 Brown Swiss bulls was used to extend the training set, as Fleckvieh is the major breed in Austria and Brown Swiss has common history with the Tyrol Grey. Breeding values (EBV), deregressed breeding values (dEBV) and their corresponding reliabilities for 10 major production and functional traits in Austria were provided by *Zuchtdata EDV- Dienstleistungen GmbH*, Austria. The deregression procedure of the EBVs removed the contribution of relatives other than daughters to the breeding value, based on the methodology of Garrick et al. (2009).

GEBV was estimated by fitting a polygenic effect assuming that every marker has a constant variance (GBLUP) (Meuwissen et al., 2001) i.e., assuming that each marker explains an equal proportion of the total genetic variance (σ^2^_g_). The GBLUP model was:

$$y=1_{n}\mu +Zg+e$$

*y* = EBV or dEBV;

*1_n_* = vector of 1 s;

μ = overall mean;

*Z* = design matrix allocating records to breeding values;

*G* = vector of random additive genetic effect using the genomic relationship matrix (G)

coming from *N*(0, *G*σ^2^_*g*_);

*e* = vector of random residual errors *N*(0, *R*σ^2^_*e*_), where *R* was diagonal matrix with weight calculated as *r*^2^/(1 − *r*^2^)

To study the predictability of the above model, three strategies were used to group the animals into reference and validation sets, with main focus on the genomic evaluation of the Tyrol Grey breed.

1. Single breed scenario: Only Tyrol Grey bulls were used. The validation sets consisted from young bulls born after 2003, with the older Tyrol Grey bulls in the reference set.
2. Multi-breed scenario: The validation sets consisted from young Tyrol Grey bulls born after 2003, the reference sets included the rest of the Tyrol Grey bulls, as well as the Brown Swiss or the Fleckvieh or both Brown Swiss and Fleckvieh.
3. Across breed scenario: All Tyrol Grey bulls were put into the validation set. The reference set consisted of the Fleckvieh and/or Brown Swiss.

**Figure 1:**
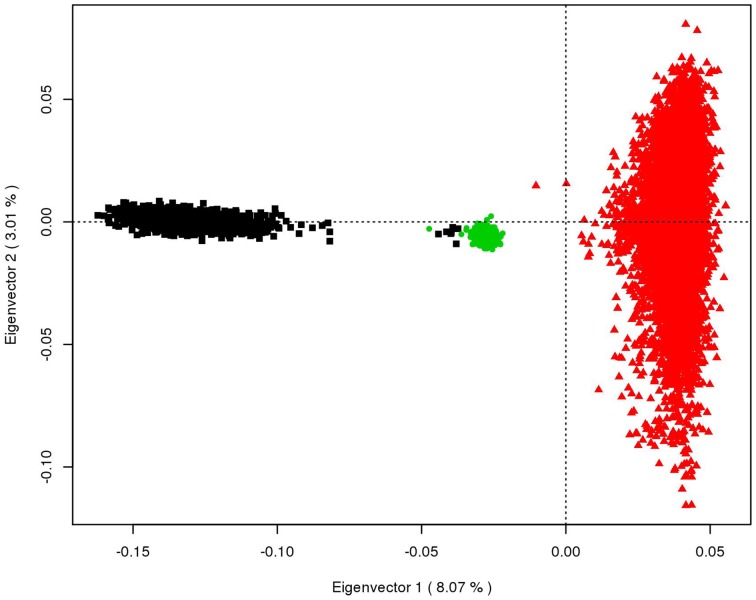
**Principal component analysis of the Tyrol Grey (in green), Brown Swiss (in black), and Fleckvieh (in brown) breeds; The amount of explained variance by the first two eigenvectors is shown in brackets**.

To be able to discuss the results obtained from the multi-breed and across breeds' scenarios, Eigen vectors, and values are computed on an estimated genomic relationship matrix with the three breeds. Principal component analysis plots are provided in Figure 1.

Prediction accuracy was measured as the correlations between the resulting GEBVs and pseudo-phenotype EBV. Bootstrapping procedure (sampling with replacement) was used to calculate the standard error of the correlation between the GEBV and EBV. The estimated GEBV were bootstrapped 10,000 times (this value appeared to give stable results) and the bootstrap GEBVs are correlated to the EBVs. The standard errors were calculated from the 10,000 estimated accuracies. This procedure gives us a fair estimate of the degree of dispersion of the estimated correlations. Although other cross validation procedure like random splitting procedures could have been employed, we chose to use forward prediction which is more relevant in breeding. In addition, limited number of individuals in the validation set also supports the idea of bootstrapping to calculate standard errors of the correlation estimates.

## Results

### Linkage disequilibrium and effective population size

**Figure 2:**
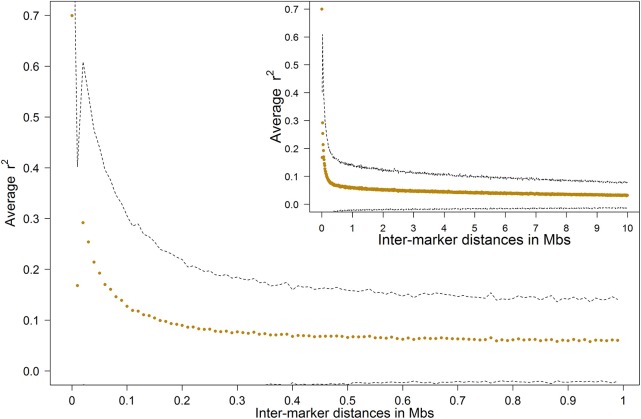
**Average LD decay in the Tyrol Grey population, dashed lines show the standard deviation boundary**.

LD was computed as squared correlations (*r*_*LD*_^2^) for all SNP pairs within chromosomes. The LD was high for markers close to each other, but decayed quickly with growing inter marker distance (Figure 2). The *r*_*LD*_^2^ was around 0.7 for very short inter-marker distances below 10 kb, but was 0.1 for marker distances at ~150 kb. After this it followed by a moderate decay until 10 Mb (*r*^2^_*LD*_ = 0.03). The average inter-marker distance in our study was ~75 kb, with average *r*_*LD*_^2^ of 0.192 ± 0.254 for adjacent markers.

**Figure 3:**
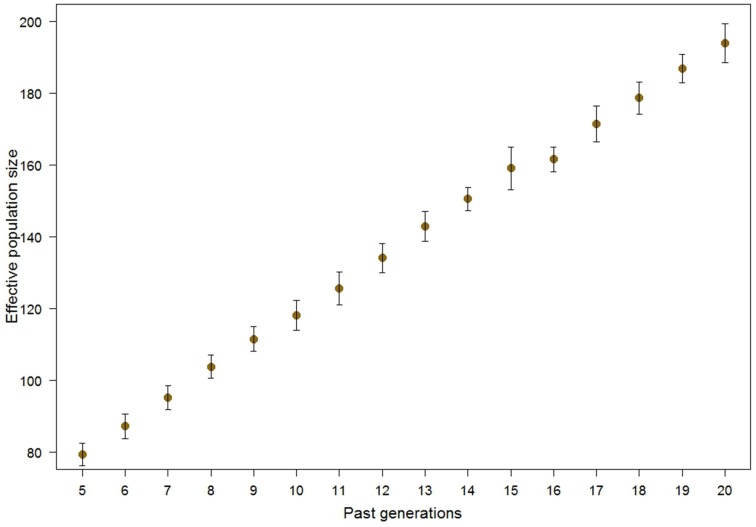
**Means and standard deviations for the historical Ne_**LD**_ in Tyrol Grey cattle, accounting for mutation and ratio of genetic per physical distance of 1.25**.

The LD was used to estimate historical Ne (Figure 3). The method of calculation allows varying genetic distance and mutation occurrence, leading to slightly different results. In our case we calculated historical Ne based on the most likely scenario, i.e., considering mutations to occur and genetic distance per unit of physical distance (cM/Mb) of 1.25, according to Arias et al. (2009). The Ne_LD_ was around 200 about 20 generations in the past and declined to about 80 in the following 15 generations. The standard deviations of estimates show the uncertainty caused by slightly differing results from multiple LD windows pointing to the same generation.

### Runs of homozygosity

**Figure 4:**
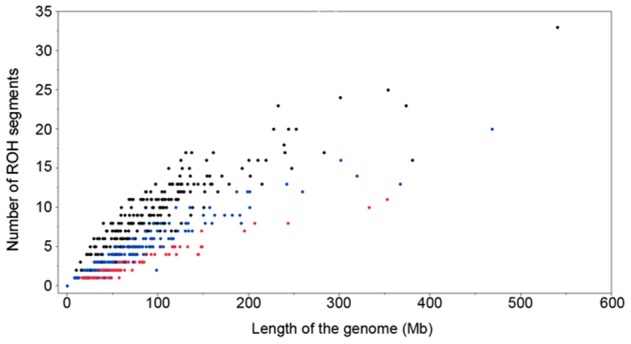
**Number of ROH segments and the length of the genome covered by ROH segments (minimum ROH length set to 4 Mb in black, 8 Mb in blue and 16 Mb in red)**.

There was a considerable difference among animals in number of ROH segments and the length of the genome covered by these ROH segments (Figure 4). For example when there was only a single ROH segment, this could be 8 to 60 Mb long. The cumulative length of the ROH segments of 60 Mb could be due to a single ROH segment, or the sum of 10 smaller ROH segments (Figure 4). Similar distributions were observed for other animals, with higher differences between total lengths of homozygous regions as the number of ROH increased. The age of inbreeding is defined as the time to the common ancestor and is quantified with the length of the ROH segments. Thus, the minimal ROH length of 4 Mb implies a common ancestor dating 12 generations in the past. Similarly the minimal ROH length of 8 and 16 Mb implies a common ancestor dating 6 and 3 generations ago, respectively.

**Figure 5:**
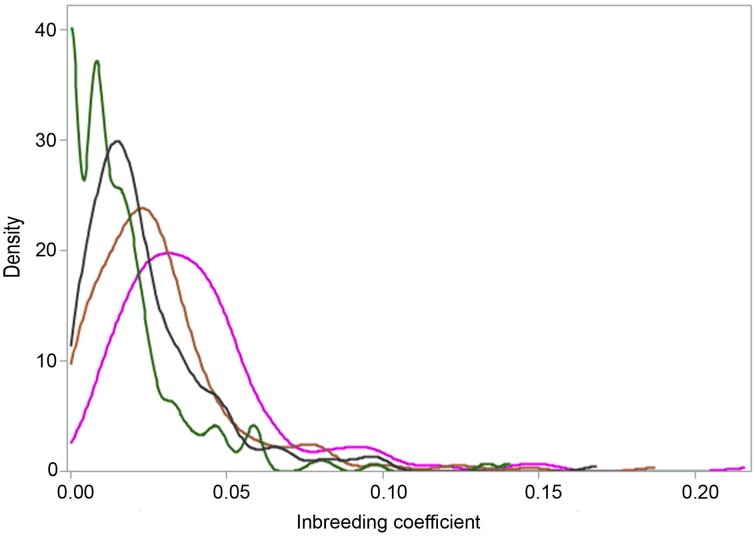
**Distributions of three ROH (*F*_**ROH>4 Mb**_ in magenta; *F*_**ROH>8 Mb**_ in brown and *F*_**ROH>16 Mb**_ in green) and one pedigree (*F*_**PED**_ in black) inbreeding coefficients for the Tyrol Grey cattle**.

The summary statistics for the three ROH (*F*_ROH>4 Mb_, *F*_ROH>8 Mb_, and *F*_ROH>16 Mb_) and one pedigree (*F*_PED_) inbreeding coefficients are presented in Table 1 and in Figure 5. Considering pedigree depth of the Tyrol Grey population, the values obtained are in agreement with the assumption that *F*_ROH>4 Mb_, *F*_ROH>8 Mb_, and *F*_ROH>16 Mb_ correspond to the reference ancestral population where the common ancestors are approximately considered to be 12, 6, and 3 generations remote as well as with values obtained in other populations (Ferenčaković et al., 2013). Animals with extreme pedigree inbreeding, for example after the threshold where *F*_PED_ > 0.05, were precisely identified by *F*_ROH>8 Mb_. The two peaks in Figure 5 are caused by the fact that *F*_ROH>16 Mb_ values cannot be smaller than 0.006, i.e., 16 Mb divided by genome covered by SNPs on the chip.

**Table 1:** **Levels of inbreeding (F) with lower and upper 95% confidence intervals (L95CI, U95CI), change of inbreeding per generation (ΔF) and inbreeding effective population size [Ne, with Ne = 1/(2ΔF)]**.

**Statistic**	**ROH > 4 Mb**	**ROH > 8 Mb**	**ROH > 16 Mb**	**Pedigree**
F	0.040	0.029	0.016	0.024
L95CI	0.036	0.025	0.014	0.021
U95CI	0.044	0.032	0.019	0.027
ΔF	0.004	0.003	0.001	0.005
Ne	125	186	370	102

The relationship between the number of ROH segments and the length of the genome covered by ROH is shown in Figure 4. A considerable difference among animals has been found in number of ROH segments and the length of the genome covered. Animals with the same total ROH inbreeding (*F*_ROH>4 Mb_) might have a different number of ROH segments but with different lengths, which is a consequence of the different distances from the common ancestors.

Ne_ROH_ derived from change of inbreeding levels per generation (ΔF) is lowest when estimated from pedigree information and increases with restriction to longer ROH segments (see Table 1). The very high Ne_ROH_ (Ne_ROH>16 Mb_ = 370) indicates strict avoidance of close inbreeding (like half sib, parent-offspring or first cousin mating) by the Tyrol Grey cattle breeders.

**Figure 6:**
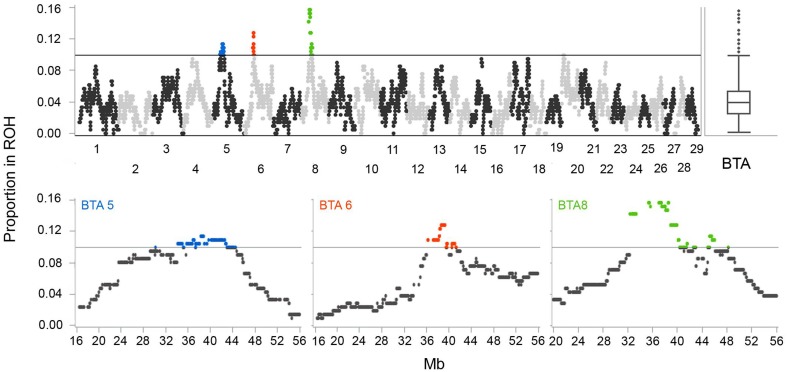
**Autozygosity islands, regions with extreme ROH frequency in Tyrol Grey cattle (minimum ROH length set to 4 Mb)**.

We have identified three regions with outlying ROH frequencies (4 Mb threshold) on chromosomes 5, 6, and 8 (Figure 6). Regions with increased ROH frequencies, the highest genomic autozygosity, are most likely consequences of selection as shown by Kim et al. (2013) and in computer simulations by Curik et al. (2002). The first region with the highest signal on BTA 8 starts at position 32,450,361 (BTB-00258020) and ends at position 46,041,080 (SNP BTA-28204-no-rs). Second region positioned on BTA 6 starts at position 36,277,967 (BTA-97637-no-rs) and ends at position 41,123,393 (SNP BTB-00406718). Finally, the region on BTA 5 starts at position 34,101,843 (BTB-01495784) and ends at position 42,918,584 (BTA-73464-no-rs). There are 147, 23, and 38 genes with known or unknown function within the signal regions on BTA 8, 6, and 5, respectively.

### Genomic selection

Genomic breeding values for Tyrol Grey bulls using major production and functional traits were computed. For the production traits the breeding values (EBV) and deregressed breeding values (dEBV) for milk yield, fat yield and content, protein yield and content were considered. For functional traits EBVs and dEBVs for longevity, persistency, maternal fertility, somatic cell count, and milking speed were included.

**Figure 7:**
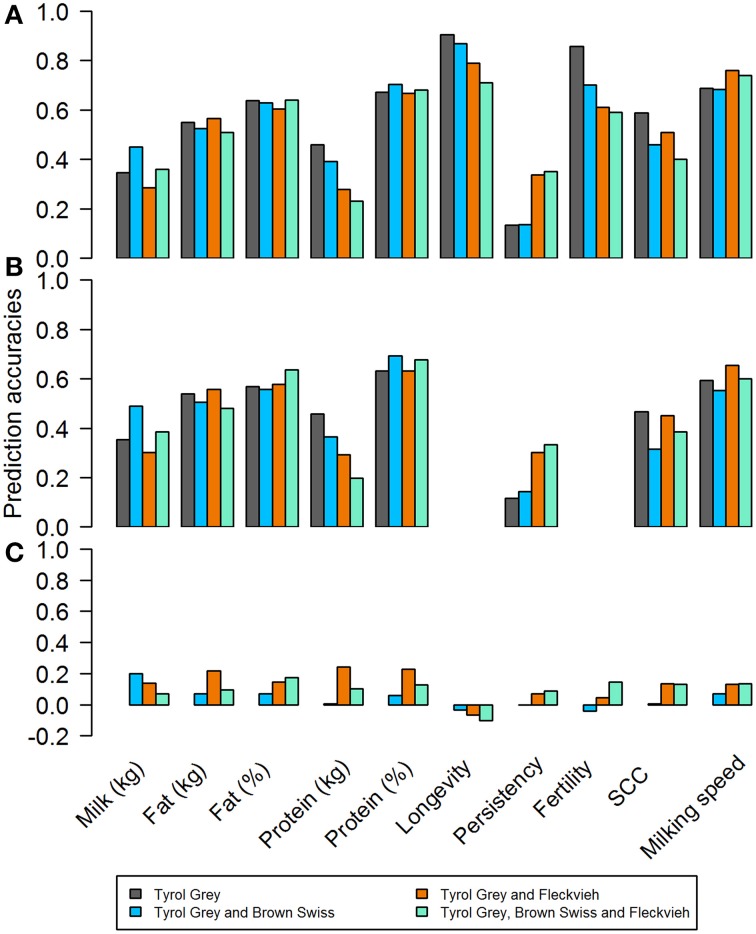
**Correlations between estimated breeding values and genomic breeding values based on (A) EBV for single and multi-breed reference sets; (B) dEBV for single and multi-breed reference sets; (C) EBV for across breed reference sets**.

Single breed evaluations were used with only old bulls and young bulls born before 2003 as reference population. The validation animals consisted of bulls born after 2003. The number of animals in the validation set differed based on the trait, but in general they were between 36 and 49 when EBVs were used, and between 29 and 42 when dEBVs were used as response variable. The results are shown in Figures 7A,B. In general the average accuracies ranged from 0.13 to 0.91. Accuracy for the production traits in kg (fat kg, protein kg) was lower than using their direct counterpart measured in percent (fat% and protein%). This has largely been attributed to higher heritabilities for fat and protein content, compared to fat and protein production. For almost all functional traits however, the correlations were much higher compared to any of the previously reported results in the literature. In fact on average they were even higher than that of the production traits. The follow up bootstrapping generated large standard errors for all traits.

In order to improve accuracies for production traits, a multi-breed approach was undertaken by adding genotypes to reference population from other breeds. Just like in the single breed scenario, validation individuals consisted of bulls born after 2003. In theory the increase in the size of reference population should increase the prediction accuracy. However, the gains and losses in accuracies varied considerably, depending on the trait, when either Brown Swiss, Fleckvieh or both populations) were added to the Tyrol Grey reference. In general, for all functional traits, adding other breeds resulted in lower accuracies, except for persistency.

When EBVs were used as response variable (Figure 7A) the impact of adding Fleckvieh into the reference set was favorable for persistency. For all other traits the results did not improve or were even worse with mixed reference sets. The benefits, if any, were not consistent across traits, showing a different pattern for each trait. The longevity and maternal fertility traits could not be evaluated due to lack of bulls with deregressed breeding values with reliability over 0.3 (Figure 7B).

An additional scenario to predict GEBV of Tyrol Grey bulls from another breed (Fleckvieh and Brown Swiss bulls) was studied. Unlike the multi-breed approach, an across breed scenario meant that, the reference population to estimate marker effect were either Fleckvieh or Brown Swiss bulls, while the validation set was the entire population of Tyrol Grey bulls. The correlations between the EBVs and estimated GEBV_EBV_ were very low (Figure 7C). In general, the correlations were somewhat higher with Fleckvieh bulls in the reference set, but still remained below 0.25 in all cases. For longevity, predicting GEBVs from both estimates of marker effects from Brown Swiss and Fleckvieh resulted in negative accuracies. Moreover, the accuracies obtained in with this scenario were lower than that of the single breed or multi-breed approach.

Bootstrapping was used to assess the degree of confidence in the GEBV accuracies. It showed very wide confidence intervals for estimated GEBV for almost all traits (Table 2). Contrary to expectations, the confidence intervals for both longevity and fertility remained very high.

**Table 2:** **Mean accuracies of GEBVs computed from EBVs and dEBVs from the single breed scenario and their 95% confidence intervals computed based on 10,000 bootstrap samples**.

**Traits**	**r(EBV,GEBV)**			**r(dEBV,GEBV)**		
	**Mean**	**Lower CI**	**Upper CI**	**Mean**	**Lower CI**	**Upper CI**
Milk yield	0.345	0.027	0.675	0.354	0.050	0.668
Fat yield	0.549	0.349	0.755	0.539	0.335	0.755
Fat content	0.637	0.403	0.902	0.569	0.311	0.864
Protein yield	0.460	0.205	0.727	0.458	0.193	0.741
Protein content	0.673	0.493	0.869	0.632	0.445	0.830
Longevity	0.906	0.859	0.950	–	–	–
Persistency	0.133	−0.101	0.364	0.117	−0.139	0.373
Fertility	0.858	0.777	0.944	–	–	–
SCC	0.589	0.383	0.804	0.468	0.188	0.747
Milking speed	0.688	0.521	0.856	0.594	0.343	0.849

In addition to estimating the correlation between GEBVs and EBVs, we also computed the correlation between GEBVs and parent averages (Table 3). High correlations signify that the estimated GEBVs only predict the part of EBVs estimated as parent averages [0.5 (*EBVsire* + *EBVdam*)]. The correlations between the GEBVs and the parent averages for EBVs and dEBVs were very moderate to high. These correlations are the highest for longevity and fertility, indicating that the high GEBV accuracies were driven by parent averages. In other words, there is no advantage of GEBVs over parent averages for longevity and fertility, and relatively little advantage for other traits in the Tyrol Grey population.

**Table 3:** **Average reliabilities of validation animals and correlations between parent averages based on EBV and GEBV/PA based on dEBV and GEBV**.

**Traits**	**Average EBV reliability**	**r(PA-EBV, GEBV-EBV)**	**r(PA-dEBV, GEBV-dEBV)**
Milk yield	0.74	0.67	0.64
Fat yield	0.74	0.77	0.73
Fat content	0.74	0.79	0.71
Protein yield	0.74	0.73	0.69
Protein content	0.74	0.82	0.76
Longevity	0.26	0.81	NA
Persistency	0.70	0.39	0.35
Fertility	0.30	0.92	NA
SCC	0.58	0.75	0.62
Milking speed	0.57	0.88	0.86

## Discussion

### Genomic analysis in tyrol grey cattle

Traditionally the research interests in small and endangered populations are in genetic diversity parameters and breed conservation efforts. The justification is to describe breeding resources which might be important for coping with future needs and for facilitating the sustainable use of marginal areas (Toro et al., 2009). Microsatellites were a popular tool to describe genetic diversity (Baumung et al., 2004). In addition to microsatellites, SNP markers have been used to describe genetic diversity via parameters like allelic richness, heterozygosity/homozygosity levels (Makina et al., 2014), or LD and the associated Ne_LD_ (Hill, 1981; Hayes et al., 2003; Tenesa et al., 2007; Medugorac et al., 2009; Flury et al., 2010).

LD, measured as the correlation between alleles, is a fundamental concept in molecular genetics, while a large number of genomic methodologies are highly dependent on it (McKay et al., 2007; Pérez O'Brien et al., 2014). A typical LD pattern was observed in our study, with high LD for markers close to each other, quickly decaying with increasing inter-marker distance. Similar patterns were observed also in other studies (de Roos et al., 2008; Flury et al., 2010; Qanbari et al., 2010). In addition to the genome wide scale it is possible to utilize the LD information on the gene level. An example of this approach was the description of the entire genetic variability of a meat tenderness gene with only 16 polymorphic SNPs and 18 haplotypes in three French cattle breeds (Marty et al., 2010).

LD can be used to calculate Ne_LD_ (Hill, 1981), even when the pedigree information is missing or it is incomplete. As the Ne size is sometimes used as a criterion to determine the endangerment status of a breed and thus always of interest. The Ne_LD_ relies on assumed impact of mutation and recombination distance (McEvoy et al., 2011), thus neglecting mutation rate or approximating the recombination distance to 1 Mb ≈ 1 cM leads to different outcomes (Corbin et al., 2012).

We note here that, calculation of Ne based on genomic data is deemed controversial. The Ne_LD_ was nearly the same in two Finnish pig populations when compared to pedigree data (Uimari and Tapio, 2011), much lower in a Swiss cattle breed (Flury et al., 2010) and strongly biased upwards in a Spanish pony population (Goyache et al., 2011). Simulation studies showed a downward bias for Ne_LD_ (Sved et al., 2013). As there are several theoretical conflicts in the estimation procedure, extreme caution is advised when calculating Ne_LD_ (Goyache et al., 2011; Corbin et al., 2012).

Several other methods were developed to overcome some of the limitations of Ne_LD_. The most popular approaches are chromosome segment homozygosity (Hayes et al., 2003) and calculation of Ne based on inbreeding rate per generation calculated from ROH (MacLeod et al., 2013, 2009; Curik et al., 2014). Here we have presented the estimation of several Ne_ROH_ depending on the three ROH length thresholds. The method is direct extension of the estimation of Ne based on pedigree inbreeding coefficient and has not been evaluated empirically. While the values obtained for *N*e_ROH>4 Mb_ are close to, although somewhat higher, Ne_LD_ and Ne_PED_ broader empirical evaluation of the method is required for its comprehensive understanding. We would like to point out that Ne_ROH_ and Ne_LD_ are two conceptually different estimates that can supplement and/or substitute Ne_PED_ estimates and provide useful information for the conservation management of a population in question. The Ne based on genomic information could be directly applied, for example in determining the risk status of breeds, as the current method used by FAO relies solely on number of male and female animals.

In the genomic selection era, SNP information is predominantly used for breeding value estimation. The popularity of genomic selection (Meuwissen et al., 2001; Hayes et al., 2009) resulted to the routine genotyping of young bulls in several large breeds (e.g., Holstein, Fleckvieh). These genotypes accumulate to an ever growing reference population which is subsequently re-used to estimate SNP effects to improve the accuracy of GEBV (Van Raden et al., 2011a). These large reference populations allow the genomic selection to be so successful (Misztal, 2011). Given the small population size of the Tyrol Grey cattle, and many other small breeds, the size of the reference population will not be high enough to meet standards of large breeds, especially in reference population size. In order to increase the reference population size other breeds are sometimes added to the breed of interest (multi-breed) or used entirely alone (across breed) to calculate marker effects in genomic evaluations.

Estimated breeding values and deregressed breeding values for a range of traits were used to assess the feasibility of genomic selection is the Tyrol Grey breed. Surprisingly high accuracy of GEBV was obtained in the single breed evaluations (Figure 7A) and especially for longevity and fertility. Based on our criteria of discarding records with dEBV reliability below 0.30, GEBV were not estimated for longevity and fertility when using dEBVs as pseudo-phenotypes. The reason for the high accuracy of prediction for GEBV_EBV_ was that, reliabilities of EBVs for young bulls were low. With low reliabilities, EBVs were similar to parent averages. This was affirmed by the high correlation between EBVs and parent averages (Table 3). Similarly large reliabilities were reported by Morota et al. (2014) when GEBV were correlated to low reliable EBVs.

In small breeding populations, the opportunities to obtain a sufficiently large number of daughters to generate highly reliable EBVs in a progeny testing scheme are limited. The problem is compounded especially for lowly heritable traits. For example, with trait heritability of 0.34 (milk yield), about 100 daughter records are need achieve reliabilities of 0.90. With heritability of 0.12 for longevity and 0.02 for fertility in our data set, about 300 and 1840 daughter records would be need. As shown in this study, predictive ability of a forward prediction scheme using young bulls as validation set was unusually high from PA driven EBVs. Lower reliabilities has been reported for the same traits with similar heritability in other large population breeds such as Fleckvieh (Ertl et al., 2014) or Holstein (Olson et al., 2012). The results from the study affirms the idea that, validation animals should have reliable EBVs if predictive ability is computed based on the correlation between GEBV and EBV. An alternative to this problem would be to use a single-step GBLUP approach (Legarra et al., 2014). Reliabilities would be computed based on the inverse of the diagonal element of the MME (Henderson, 1975). Reliabilities computed using the single step GBLUP approach could be compared to reliabilities of parent averages. Potential benefit of use of genomic information could be directly measured.

Multi-breed reference populations for genomic prediction are highly dependent on the LD and structure and genetic distance between breeds. The accuracy of genomic prediction could be substantially improved when the breeds are genetically very close or when animals of the same breed from multiple countries are pooled (Lund et al., 2014). Also using Bayesian variable selection instead of the BLUP approach could be beneficial in case of more distantly related breeds (Erbe et al., 2012; Bolormaa et al., 2013; Zhou et al., 2014). Lower accuracies in multi-breed genomic evaluation can be attributed to extent and differences in LD between markers and QTL (Goddard and Hayes, 2009), phase and allele substitution effects of QTLs (Spelman et al., 2002; Thaller et al., 2003).

In order to demonstrate the across breed genomic evaluation in Tyrol Grey cattle the German-Austrian Fleckvieh and Brown Swiss genotype pools were used in the evaluation. Using the large reference population composed of these two breeds to estimate GEBV was not successful. As shown in Figure 7C, the accuracies were very low for all traits. The accuracies have improved when part of the Tyrol Grey bulls were included into the reference set. This multi-breed reference however, did not have a clear advantage over the accuracies from the small breed reference set, similarly to Karoui et al. (2012).

A large population size is generally perceived as a requirement to estimate reliable GEBV, as we highlighted in the introduction of this paper. When the population is below of a critical mass the EBVs will be driven by parent averages, therefore genomic selection techniques will bring little new information into genetic evaluation of small breeds, as demonstrated in our paper. On the other hand, if reliable pedigrees are not available in a certain breed, i.e., no conventional breeding value estimation can be done, the breeding values estimated based on genomic data are a secure way to improve the breed.

### Additional uses of genomic data for management of small and endangered breeds

Even if genomic selection methods produce uncertain results in small breeds, there are a number of other reasons why a routine genotyping of the population would be beneficial. The identification of relatedness and inbreeding levels in the population has one of the biggest advantages from the practical perspective. The genomic relationship matrix can uncover family structures and infer relatedness within the population (Supplement Figure 1), even if the pedigree information is missing or it is incomplete (Calus et al., 2011). The correctness of the existing pedigrees can be verified comparing genomic information, e.g., by checking for Mendelian inconsistencies to identify incorrect parent-offspring relationships.

Similarly to the genomic relatedness it is possible to calculate the inbreeding coefficient. Compared to non-genomic approaches, here the knowledge of the pedigree is not needed, and so equally good results can be produced for animals, whose pedigree is dubious, incomplete, or entirely missing. Runs of homozygosity extend the analysis of relatedness between two individuals by identifying long homozygous segments, supposedly coming from the same ancestor somewhere in the past, inferring the individual's inbreeding coefficient. Based on the length of the segments it is possible to identify the number of generations to the common ancestor. In this study, the mean level of inbreeding estimated from three different ROH lengths (*N*e_ROH>4 Mb_, *N*e_ROH>8 Mb_, and *N*e_ROH>16 Mb_) ranged from 0.016 to 0.029 and were comparable to other studies. In addition, there were only four individuals with outlying inbreeding coefficients higher than 0.125, indicating that potential risks could have been even more reduced with genomic information available. The utilization of genomic information to control inbreeding as well as to reduce early embryonic loss or appearance of congenital genetic defects due to recessive haplotypes in homozygous state (see more detailed discussion below) seems promising.

Crossbreeding is a very common strategy to increase the productivity of a breed or to introduce a desirable quality from another breed. The levels of crossbreeding are traditionally computed based on pedigree information. The pedigree approach assumes that the genetic composition of individuals with the same type of ancestry information is equal. This assumption does not hold however, as recombinations alter the composition of ancestral chromosomes, resulting into different admixture levels (Bryc et al., 2010). The Girolando cattle for example were bred to achieve a 5/8 of Holstein and 3/8 of Gir cross. Based on the pedigree information the expected Holstein admixture level is 62.5%. The real admixture levels based on SNP data can vary as much as 49–85% (Orazietti et al., 2014, unpublished). The adaptability of breeds can be also increased by producing optimal composites for a specific region. For example, introducing the alleles that are responsible for the trypanotolerance in Baoule cattle into the genomes of the trypano susceptible zebu populations in Burkina Faso would be a great advantage (Soudré et al., 2013). In other small populations the crossbreeding with large commercial populations could be a concern due to the loss of purebred stock. In all cases SNP chip data provides reliable estimates of the admixture levels which facilitates the selection of the desirable genotypes for breeding purposes (Frkonja et al., 2012). Furthermore, the genomic information could be used to purge the foreign genome from a small population (Amador et al., 2014).

A less frequent, but a much more critical utilization of genomic data is detection of lethal or sub-lethal genotypes. The obvious case is when a disorder is found in a population and an attempt is made to discover its source and genetic background by *ad hoc* genotyping of affected individuals. A very good example for this *ad hoc* approach was the disorder similar to bovine progressive degenerative myeloncephalopathy (weaver syndrome) in the Tyrol Grey population. As the purebred population is small, the disorder would have had a devastating effect. The region with the causative mutation was identified combining homozygosity mapping (Charlier et al., 2008) and other genome wide association techniques in 14 affected and 27 control animals. More detailed analysis allowed pinpointing the causal mutation in the mitofusin (MFS2) gene. Routine genotyping of breeding animals identifies any carriers and will purge the population from this mutation within a short period (Drögemüller et al., 2011).

The previous case demonstrated an efficient an identification of causal variants for a known disorder. If the disorder itself or its symptoms were less obvious however, the detection of affected animals may be much more difficult. To detect these cases it is possible to screen the whole population genotype data. Alleles with relatively high heterozygote frequency in the population, but without the occurrence of both homozygotes indicate lethal variants. Eleven candidate haplotypes were detected using this technique in the North American Holstein, Jersey, and Brown Swiss population, some of them with confirmed phenotypic effects (Van Raden et al., 2011b). Similar technique was used to identify homozygote deficient haplotypes with potentially negative effects on fertility traits in Nordic Holstein (Sahana et al., 2013) and Jersey (Sonstegard et al., 2013). In most cases the frequency of carrier animals with harmful genomic regions in heterozygous state is relatively low, but it can also be surprisingly widespread. In Finnish Red cattle a region associated with embryonic death had a frequency of 32% in the population, due to its positive effect on milk yield (Kadri et al., 2014). In general, the genotype screening allows the detection of new disorders or to confirm the causative sites of known defects. These disorders and defects can be then avoided in subsequent generations by planned mating of carriers and non-carriers, or even eradication of certain disorders from the breed by restricted usage of carrier genotypes.

## Conclusions

In a very short time, high-throughput molecular information has become a standard tool in animal breeding. Routine genotyping of the entire male population in small breeds is often not in place, although it would be feasible due to the small population size and extreme reduction in genotyping price. Our results suggest that genomic selection is not readily applicable in small breeds even with very large reference populations in a multi breed setting. There are numerous other utilizations of the genomic information however, that make routine genotyping not only beneficial but outright desirable for the management of small breeds. Apart from various genetic diversity measures, the identification of regions identical by descent instead of approximations according to the pedigree will help to better understand relatedness and inbreeding in the population. Furthermore, the pool of genotypes for the entire breed enables to continuously scan the population and allow a swift reaction in identifying carriers of lethal or potentially harmful haplotypes. The new information can be used to eliminate undesirable alleles through the mating process. Similarly, the breed proportions due to admixture could be estimated with the goal to fix a desirable ratio or to preserve the purity of the breed.

While our paper describes an example from the Tyrol Grey population, we would like to stress that the recommendations are valid for all small and endangered breeds. The genotyping of SNP markers is a mature and well understood technology, with uses that can complement, improve or even replace approaches for breed management. Therefore, we suggest the continuous collection of genotypes and their use in breed monitoring and improvement.

### Conflict of interest statement

The authors declare that the research was conducted in the absence of any commercial or financial relationships that could be construed as a potential conflict of interest.
