# Supplementary figures and images for: Genomic analysis for managing small and endangered populations: a case study in Tyrol Grey cattle
Source: Front Genet. 2015 May 13;6:173. doi: 10.3389/fgene.2015.00173 (PMC4443735; doi:10.3389/fgene.2015.00173)

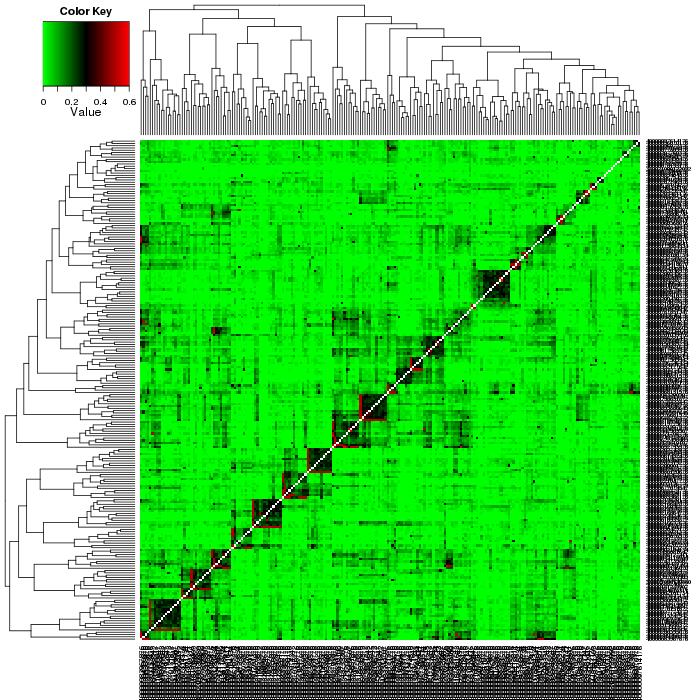

Supplement: Supplementary file 1 [file Image1.JPEG]
